# Molecular and Mechanical Cues for Somite Periodicity

**DOI:** 10.3389/fcell.2021.753446

**Published:** 2021-11-26

**Authors:** Marta Linde-Medina, Theodoor H. Smit

**Affiliations:** ^1^Independent Researcher, Palma, Spain; ^2^Department of Orthopaedic Surgery, Amsterdam Movement Sciences, Amsterdam University Medical Centres, Amsterdam, Netherlands; ^3^Department of Medical Biology, Amsterdam University Medical Centres, Amsterdam, Netherlands

**Keywords:** clock and wavefront, differential strain, scaling, somitogenesis, vertebral column

## Abstract

Somitogenesis refers to the segmentation of the paraxial mesoderm, a tissue located on the back of the embryo, into regularly spaced and sized pieces, i.e., the somites. This periodicity is important to assure, for example, the formation of a functional vertebral column. Prevailing models of somitogenesis are based on the existence of a gene regulatory network capable of generating a striped pattern of gene expression, which is subsequently translated into periodic tissue boundaries. An alternative view is that the pre-pattern that guides somitogenesis is not chemical, but of a mechanical origin. A striped pattern of mechanical strain can be formed in physically connected tissues expanding at different rates, as it occurs in the embryo. Here we argue that both molecular and mechanical cues could drive somite periodicity and suggest how they could be integrated.

## Introduction

Vertebrates are defined by the presence of the vertebral column. This characteristically segmented structure derives primarily from two pieces of mesodermal tissue located at each side of the body axis, hence the name *paraxial* mesoderm (PM). One of the first morphogenetic events during the formation of the vertebral column is the division of this tissue into regularly sized portions (i.e., somites), a process called somitogenesis (Supplementary Movie 1). Except for some exceptions (e.g., the anterior most somites in the chicken embryo) [see Supplementary Movie 3 in Dias et al. (2014)], the segmentation of the PM does not occur at once, but single somites successively detach from the anterior border at a species-specific rate until the whole PM becomes divided (Gómez and Pourquié, 2009).

Segmentation coincides with a maturation gradient along the body axis of the embryo. At the posterior region, cells are sparsely embedded in a soft, hyaluronan matrix (Straaten et al., 1990), where they have large intercellular spaces and move randomly (Bénazéraf and Pourquié, 2013). Toward the anterior region, hyaluronan is enzymatically degraded (Stern, 1984; Straaten et al., 1990), which reduces the extracellular space and hence increases the cellular interactions (Bénazéraf and Pourquié, 2013). These interactions not only occur by a higher concentration of bounding molecules like N-cadherin (Duband et al., 1987), cadherin-11 (Kimura et al., 1995), and N-CAM (Linask et al., 1998), but also by the slender protrusions that stick out of the cells themselves (Shawky et al., 2018).

Toward the anterior end, the peripheral cells of the PM connect to the fibronectin matrix and form an epithelial layer (Martins et al., 2009). Under this mesenchymal-to-epithelial transition, the peripheral cells become polarized: integrins establish a physical connection to the underlying matrix at their basal side, but not at their apical sides, which face the mesenchymal inner cells (George et al., 1993; Bryant and Mostov, 2008). This will lead to the formation of a monolayer. Adjacent epithelial cells attach to each other by cadherins (Halbleib and Nelson, 2006), tight junctions (Baum and Georgiou, 2011), and gap junctions (Nelson et al., 2006), which further increases the coherence of the epithelial layer along the posteroanterior axis. Importantly, this epithelial layer physically connects the PM with the surrounding tissues by means of fibronectin fibrils (Martins et al., 2009).

During the formation of a somite in the anterior region, a fissure appears in the mesenchyme that connects with an already formed cleft in the epithelial layer (Takahashi and Sato, 2008; Adhyapok et al., 2021). Mesenchymal cells at the fissure undergo a mesenchymal-to-epithelial transition, which leads to the formation of a physical boundary that is further stabilized by the deposition of extracellular matrix (fibronectin) (Dahmann et al., 2011). These events culminate in the formation of a somite, i.e., an epithelial sphere with a small cavity (the somitocoele) filled with untransformed mesenchymal cells (Martins et al., 2009). By the time a new somite detaches from the unsegmented PM, the maturity gradient has progressed toward the tail, so cells now occupying the anterior region are ready to budding off.

Somitogenesis is a time regulated process: somites form at a precise time interval, which is species-specific (for a review see Saga and Takeda, 2001). Another characteristic feature of this morphogenetic process is scaling: the number of somites is independent of body size, i.e., embryos with altered body lengths will still form the species-specific number of somites (Cooke, 1975; Ishimatsu et al., 2018), a phenomenon referred to as *scale invariance* (Umulis and Othmer, 2013). This scaling mechanism assures that the whole vertebral column will form in spite of perturbations in body length. The segmentation of the PM into regularly sized and spaced somites will contribute to the formation, for example, of a functional vertebral column. The question is: which mechanism underlies somite periodicity?

## The Molecular Approach

In the 70s, Cooke and Zeeman proposed a theoretical model of periodic tissue segmentation that still prevails in the field (Cooke and Zeeman, 1976). It is based on the existence of two components whose interaction leads to the formation of periodic structures: (1) a genetic oscillation called the clock, i.e., a gene that is expressed periodically by cells, (2) a wavefront of cell maturation. The wavefront divides the PM primarily into a posterior and an anterior region formed by immature and mature cells, respectively. Only cells at the mature state are capable to form a somite. The wavefront moves from head-to-tail at pulses induced by the clock, i.e., the progression of the wavefront is stopped when the clock is “on,” and restarts when the clock is “off.” A somite forms when mature cells turn their clocks “on” and undergo a “catastrophic event” leading to somite formation (detachment from the unsegmented PM, cohesion and stabilization of the detached piece).

**FIGURE 1:**
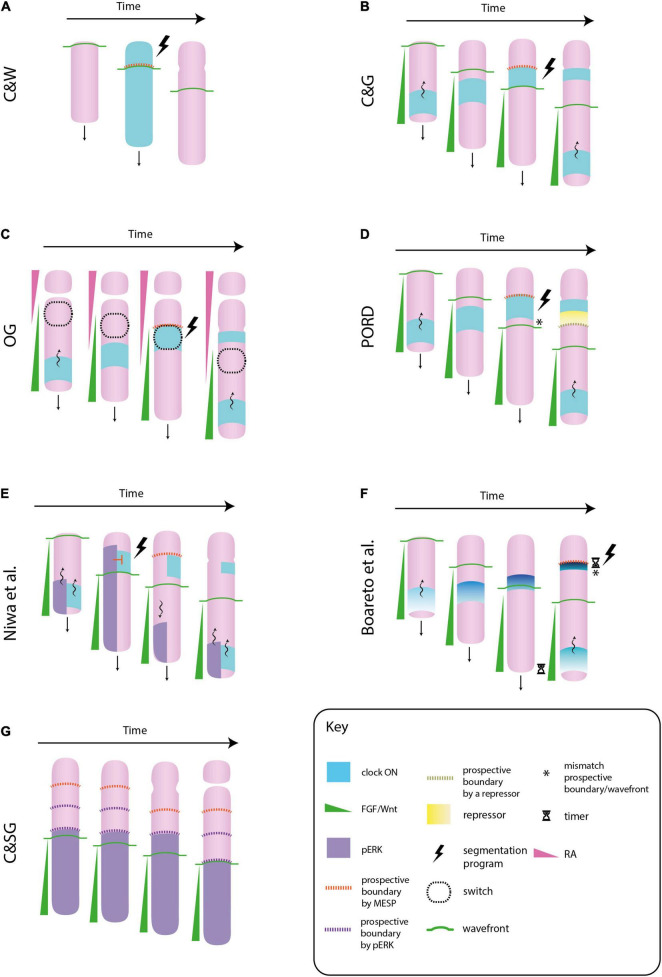
Different molecular models of somitogenesis. In the original clock and wavefront model (C&W), cells oscillate in phase, i.e., the whole paraxial mesoderm would switch “on” or “off” the clock gene at once. The interaction between the clock and the wavefront transforms a whole block of mesenchymal cells into a somite (an epithelial sphere) **(A)**. In the clock and gradient model (C&G), the wavefront is formed by a FGF gradient that progressively regresses toward the tail. Cells synchronize their clocks in a way that they form a stripe of gene expression that sweeps from tail to head. The gradient specifies the position at which the clock slows down and triggers the segmentation program. In contrast to C&W, the wavefront and clock mechanism specifies not a whole somite, but its boundary **(B)**. In the opposing gradients (OG) model, morphogen gradients in opposite directions would form a bistability window in which cells will suddenly change from immature to a mature state if they switch “on” their clocks. This allows the formation of sharp boundaries of gene expression, as those observed in the paraxial mesoderm **(C)**. In the progressive oscillatory reaction-diffusion (PORD) model, prospective boundaries are specified by a repressor emanating from the last formed clock stripe, independently of the FGF gradient (i.e., the gradient does not provides positional information) **(D)**. In the model proposed by Niwa et al. (2011), ERK, a downstream component of FGF signaling, also oscillates. When the ERK oscillation arrives at the anterior region, it inhibits the activation of the segmentation program in mature cells that express the clock. When this oscillation regresses toward the tail, this inhibition is released and cells activate the formation of a somite boundary. In this model the role of FGF signaling is not played by a gradient of positional information, but by the oscillatory behavior of one of its components **(E)**. In Boareto et al. (2021) model the FGF/Wnt gradient does not directly mark the location of a new boundary, however these posterior signals need to be degraded for the intensity of the clock signal to increase (note how the intensity and the thickness of the clock signal decreases along the posteroanterior axis). This increase will trigger the segmentation program. The FGF/Wnt would not provide positional information, but it would regulate the timing of somite formation by means of FGF/Wnt decay rate **(F)**. In the clock and scaled gradient (C&SG), prospective somite boundaries are set by the wavefront alone, by means of a stepwise regression of its downstream component ERK. The role of the clock would be to reinforce this boundaries **(G)**.

Regularly spaced and sized somites will periodically form as the wavefront progresses toward the tail and the clock oscillates at a constant period. This model has been called the *clock and wavefront* model (C&W) (Figure 1A). According to it, a wavefront that progresses toward the tail at a higher speed or a clock that oscillates with a longer period will lead to the formation of larger somites, as more cells will be mature (i.e., responsive) by the time the clock switches “on.” On the other hand, a slower wavefront or a clock with a shorter period will lead to the formation of smaller somites, as by the time the clock is “on” a reduced number of cells will be mature. Therefore, the regulation of these two parameters would assure the formation of the appropriate number of somites in case of perturbations in body length.

Subsequent molecular studies revealed the existence of both, oscillatory genes (Palmeirim et al., 1997) and morphogen gradients in the PM (Dubrulle et al., 2001), as postulated by Cooke and Zeeman (1976). Palmeirim et al. (1997) showed that the gene *c-hairy* oscillates in the PM of the chicken embryo. However, they found that cells do not oscillate in phase, as assumed by the C&W: at a specific time point some cells have their clocks “on,” whereas others have it “off.” The authors showed that cells are synchronized in such a way that they generate a stripe of gene expression that travels from tail-to-head. When one stripe reaches the anterior region, a new one starts at the posterior region. They also showed that the period of this oscillation—the time it spends to travel from the tail to the anterior extreme –, coincides with the period of somite formation, i.e., each time the oscillation reaches the anterior region, a new somite detaches. Dubrulle et al. (2001) found a tail-to-head FGF8 gradient in the chicken embryo that seemed a good candidate for a wavefront. As expected by the C&W model, an increase in FGF8 signaling at the anterior extreme of the gradient, a perturbation that simulates a slower wavefront, led to the formation of shorter somites. Contrarily, its inhibition, which simulates a faster wavefront, led to the formation of larger somites.

These findings provided strong support to the C&W model (see also Oates et al., 2012). However, they also led to the formulation of alternative hypotheses, some of which substantially deviate from the original idea. Below, we will briefly discuss some of the most recent and divergent alternatives (for a review of earlier models see Schnell and Maini, 2000).

### A Modern Version of the C&W Model

A molecular formulation of the C&W model based on the mouse embryo is depicted in Figure 1B. The wavefront is formed by a posteroanterior gradient of FGF signaling and the clock by a periodic wave of Notch signaling that sweeps in the direction of the gradient (for a review see Saga, 2012a). High levels of FGF signaling keep mesodermal cells in a mesenchymal, undifferentiated state, whereas low levels switch them to a mature state (i.e., a somite competent state). The source of FGF is located at the tail bud, and consequently, the wavefront regresses posteriorly as the embryo elongates. The Notch oscillation slows down and forms a thin stripe at the anterior region, where FGF signaling is low. This Notch stripe activates a genetic cascade in mature cells that leads to the formation of a fissure and the subsequent epithelialization of the boundary cells, i.e., the generation of a new somite. This genetic cascade is called the *segmentation program*, and the *mesp2* transcription factor (in mice) is considered the first indicator of its activation. The anterior border of *mesp2* expression marks the location of a new somite boundary. The location in the PM in which *mesp2*—or a homologous gene—is expressed is commonly called the *determination front* (e.g., Bajard et al., 2014). The posterior border of *mesp2* is defined by FGF, and therefore, the wavefront will specify the location of somite boundaries. The timing of *mesp2* expression is determined by Notch oscillation, and thus, the clock will specify the pace of somite formation (for a review see Saga, 2012b). Like in the original C&W model, the periodicity of the clock, conjointly with the gradual regression of the wavefront, ensures the formation of regularly sized and spaced somites.

However, the generality of this model has been questioned. For example, the role of Notch in the specification of somite boundary is debated (Venzin and Oates, 2020). In zebrafish, it is generally thought that Notch is only required to synchronize clock oscillations (Özbudak and Lewis, 2008). Even in mice there is some controversy, for example, it has been shown that embryos expressing Notch intracellular domain (NICD) throughout the whole presomitic PM still form somites, which questions the role of Notch oscillation in somite periodicity (Feller et al., 2008, but see also Oginuma et al., 2010). In zebrafish, the FGF8 gradient finishes before the posterior most stripe of the MESP gene *mespb*, which means that this gradient would not directly provide positional information to start the segmentation program (Bajard et al., 2014). Some other model organisms suggest that cells are committed to form a somite boundary before the activation of MESP genes (Dubrulle et al., 2001; Akiyama et al., 2014). However, in lack of a more appropriate term, we use “determination front” to refer to the location in the PM in which MESP genes are expressed.

As it will show below, a morphogen gradient is only one mechanism by which the wavefront of maturation could be implemented. Therefore, some authors suggested to call the model depicted in Figure 1B the *clock and gradient* model (C&G) (Slack, 1983). Cinquin (2007), remarked that the C&G model differs from the original C&W in two points. First, in the C&W the clock pushes the wavefront toward the tail, whereas in the C&G the latter regresses independently of the former. Second, in the C&W model the interaction between the clock and the wavefront specifies the whole somite, i.e., the block of cells that will epithelialize, whereas in the C&G model it is only the somite boundary.

### The Opposing Gradients Model

Two more morphogen gradients have been found in the PM: (1) a tail-to-head gradient of Wnt, with a source in the tail bud, (2) a head-to-tail gradient of retinoic acid (RA), with a source in the formed somites (for a review see Aulehla and Pourquié, 2010). Their specific roles in somitogenesis are not yet well understood (e.g., Mallo, 2016). Some evidence suggest that the Wnt gradient, which presents the same spatial distribution as the FGF gradient, also plays a similar role, that is, to keep cells in an undifferentiated state and specify the location of somite boundaries (Aulehla et al., 2003, 2008; Bajard et al., 2014). The main idea is that these two signaling pathways reinforce each other and conjointly maintain mesodermal progenitors in an undifferentiated state, however, FGF alone would specify the determination front. The effect of Wnt on boundary position may be indirect by secondarily affecting FGF signaling (Wahl et al., 2007; Simsek and Özbudak, 2018). Regarding the segmentation clock, it seems that both pathways act as a permissive cue to generate Notch oscillations in the posterior PM, and consequently, they will cause its slowdown at the anterior region, where both pathways have reached low expression levels (Gibb et al., 2009; Anderson et al., 2020).

FGF and RA signaling are mutual antagonists in the PM: FGF8 inhibits RA signaling by activating the synthesis of an RA degradation enzyme (Cyp26), whereas RA inhibits FGF signaling by blocking a downstream component (ERK) (del Corral and Storey, 2004; Zhang et al., 2021). Thus, the specification of the determination front would not depend solely on FGF/Wnt, but also on the RA gradient. This has been called the *opposing gradients* model (OP). It has been suggested that FGF and RA gradients can produce a bi-stable switch in the anterior PM, which will define the determination front (Goldbeter et al., 2007). In accordance with this idea, there exist two stable cell states in the PM: (1) an undifferentiated cell state favored by “high FGF/low RA” signaling, (2) a differentiated, somite competent cell state favored by “low FGF/high RA” signaling. Cells at the posterior region are in the former, and they will remain in this stable state despite changes in FGF signaling due to body elongation, until they reach the bistability window. Once there, they go through an unstable cell state in which a supra-threshold decrease in FGF and/or increase in RA signaling will quickly, and simultaneously, push them to the second stable cell state. This signal will be provided by the segmentation clock.

The displacement of the bistable window toward the posterior end is regulated by body elongation and/or degradation of FGF components at the anterior region, which conjointly with the periodicity of the clock, will progressively segment the PM (Figure 1C). A characteristic feature of bistable switches is the formation of sharp boundaries of gene expression, as those observed in the anterior PM. This kind of boundaries are more difficult to achieve with a gradient of positional information (Jaeger and Martínez-Arias, 2009). In theory, the model could specify either the whole somite or its boundary; however, note that models that specify the whole somite would be at odds with the fact that cells at the somitocoele remain untransformed.

Experiments in zebrafish have shown that explants of the PM from 10-somite stage embryos in which formed somites—i.e., the source of RA—have been removed, still develop normal somites (Simsek and Özbudak, 2018). This suggests that RA does not play a role in the definition of somite boundary, arguing against the OG model. This result could be explained by differences in the formation of the body axis among vertebrate taxa. It has been suggested that not all somites derive from the same population of neuro-mesodermal progenitors (NMPs) (i.e., bipotent cells that give place to the spinal cord and PM), but two different populations have been distinguished (Steventon and Martínez-Arias, 2017): (1) expanding-NMPs, which give place to trunk somites, (2) depleting-NMPs, which give place to tail somites. Experiments in mice have shown that RA is necessary to form trunk somites, but not tail somites, i.e., it is required for the maturation of expanding-NMPs. The absence of somite alterations in zebrafish embryos lacking RA signaling could be explained by the lack of the expanding-NMPs population in fish (Berenguer et al., 2018).

### A Reaction-Diffusion Model

A more recent proposal, called the *progressive oscillatory reaction-diffusion* model (PORD), has challenged the role of the FGF/Wnt gradient in somite boundaries (Cotterell et al., 2015). According to the authors, the pre-pattern of gene expression that precedes somitogenesis is not the readout of a gradient of positional information, but results from a reaction-diffusion mechanism (i.e., short range cell-cell interactions). FGF/Wnt upregulation at the posterior PM is necessary to pattern this tissue: FGF/Wnt activates the synthesis of the activator of this reaction-diffusion system, and thus, it will trigger the traveling wave of clock genes that eventually patterns the tissue. However, the *graded* distribution of FGF/Wnt is dispensable, as a homogeneous distribution would be enough to generate a periodic pattern of gene expression. Still, the gradient can play a role in somite scaling, that is, to generate the same number of somites despite alterations in body length.

In PORD, the determination front is defined by a short-range repressor of clock oscillations (yet to be discovered) secreted from the last-formed Notch stripe, independently of the FGF/Wnt gradient (Figure 1D). In contrast to other models, this reaction-diffusion system can generate periodic stripes of gene expression even if the FGF/Wnt gradient does not regress posteriorly. However, downregulation of FGF signaling would be necessary for cell maturation and, therefore, for the formation of somite boundaries. As stressed by Palmeirim et al. (1998), chemical patterning of the PM is uncoupled from physical boundary formation. An important feature of this model is that it is capable of generating both, simultaneous and periodic tissue patterning by just changing one single parameter value. Contrary to posterior somites, anterior somites can form almost simultaneously (see Supplementary Movie 3 in Dias et al., 2014). The PORD model shows that, despite this remarkable difference, the same mechanism could underlie somitogenesis along the whole body axis.

### The Wavefront Also Oscillates

Traveling waves of gene expression in the PM were first discovered in members of the Notch signaling pathway (Palmeirim et al., 1997). Some studies have identified putative oscillatory behavior also in members of the FGF and Wnt signaling pathways in mouse, chicken and zebrafish (Dequéant et al., 2006; Krol et al., 2011). At present, the mouse embryo is the only *in vivo* system in which these oscillations have been confirmed (Aulehla et al., 2003; Niwa et al., 2011). The oscillation of the wavefront has led to alternative explanations of somite periodicity. For example, it has been suggested that phase shifts between two oscillatory genes, rather than the interaction between the FGF/Wnt gradient and the clock, could trigger the segmentation program in the anterior PM (Beaupeux and François, 2016). It has been observed that *Axin2* and *lunatic fringe* (*Lfng*), members of the Wnt and Notch pathway, respectively, oscillate out-of-phase in the posterior PM, whereas they do it in-phase in the anterior PM (Sonnen et al., 2018). *In vitro* experiments have shown that when these two oscillatory genes remain out-of-phase, no somite boundaries are formed, i.e., their phase shift would play a relevant role in somitogenesis. However, these explants expressed *mesp2* on time, which indicates that this phase shift would not mark somite boundary, as suggested by Beaupeux and François (2016), but it could be required at later stages to form physical boundaries.

An alternative model based on FGF and Notch oscillations in mouse has been suggested by Niwa et al. (2011). According to this model, an oscillatory wave of ERK, a downstream component of FGF signaling, blocks the activation of *mesp2* by Notch at the prospective somite boundary. This inhibition is released when the ERK oscillation regresses toward the posterior region (Figure 1E). According to the authors, the roles of FGF and Notch signaling are reversed: Notch oscillations determine the location of the next segmentation point, whereas FGF oscillations set the pace of somite formation. Note that in this model, the FGF gradient plays an indirect role in setting the somite boundary by means of its oscillatory behavior.

### The Wavefront as a Timer of Somite Formation

In the model proposed by Boareto et al. (2021) the positional information to determine a somite boundary is encoded in the properties of the clock. This model is based on the observation that the traveling stripes of the clock gene *Hes/her* become thinner and more intense as they approach the anterior border (Shih et al., 2015). According to the Boareto model (2021), cells will trigger the segmentation program when the difference in *Hes/her* expression with their neighboring cells reach a certain threshold. As the intensity of the clock signal increases from tail-to-head, this difference will be maximal close to the anterior border where new boundaries are formed. The authors have suggested that the FGF/Wnt gradient would modulate these clock properties, but this link is temporal rather than spatial (for this distinction see Clark, 2021): cells need time to degrade these posterior signals in other to increase the periodicity and intensity of their clocks, and therefore, to increase the difference in *Hes/her* expression that eventually triggers the segmentation program (Figure 1F). Consequently, FGF/Wnt gradients would be a byproduct of body elongation that are not providing spatial information for boundary formation, but they would set its timing by means of FGF/Wnt decay rate.

### A “Prior Wave” of Somitogenesis

In contrast to some models in which the gradient is dispensable to pattern the PM (e.g., Cotterell et al., 2015), in the model proposed by Akiyama et al. (2014) it plays a fundamental role in somite periodicity. The authors have shown that ERK activity in zebrafish does not regress continuously alongside the FGF gradient, but it does so in a stepwise manner. During the formation of a new somite, ERK activity remains constant despite the posterior displacement of the FGF gradient. Once the somite is formed, the anterior border of ERK activity quickly regresses to match its previous relative position with the FGF gradient. This stepwise regression of ERK activity occurs in the posterior PM, two or three somite lengths far from the determination front (Figure 1G).

Cell tracking has revealed that cells located at the border of ERK activity will form future somite boundaries, i.e., ERK activity may specify the segmentation points before the determination front. This would correspond to what Pearson and Elsdale (1979) called a “prior wave” of somite determination. It matches with the observation that incipient somites can already be morphologically recognized in the posterior PM, where cells begin to compact into blocks and the future somite boundaries are marked by little clefts in the epithelium (Bellairs, 1979; Meier, 1979; Adhyapok et al., 2021). In clock-deficient zebrafish embryos, this “prior wave” is also produced, however, the stepwise regression of ERK activity occurs at irregular time intervals, which forms somites of different sizes (Sari et al., 2018). This result reveals that ERK activity can segment the PM independently of a segmentation clock. Computer simulations have suggested that the irregular somites formed in clock-less conditions would be due to an increase in the intrinsic noise of ERK activity. The authors have suggested that the role of the clock would be to reduce this intrinsic noise, thereby assuring the formation of regularly sized somites. Importantly, this model, called the *clock and scaled gradient* (the FGF gradient dynamically scales to the length of the PM), explains somite scaling under a wide range of perturbations, which gives it a certain advantage over other models (Ishimatsu et al., 2018).

### Self-Organizing Somites

Dias et al. (2014) carried out an experiment that would challenge all the models above. The authors dissected a piece of tissue from the primitive streak (i.e., undifferentiated mesoderm) from a chicken embryo and grafted it into an extraembryonic region, far from any signaling gradient. The graft was surrounded with beads soaked in Noggin to trigger the differentiation of PM tissue (it was also cultured in Noggin for 3 h before it was grafted). The graft was capable of forming somites, i.e., epithelial spheres, with a ring of N-cadherin at their apical side, surrounded by a fibronectin matrix at their basal side, and expressing somite markers (e.g., *paraxis*). Furthermore, it did not display oscillatory expression of clock genes. However, these ectopic somites did not show the characteristic rostrocaudal polarity of somites (some genes are expressed only in one half of a somite), which will guide the formation of the vertebral column (for a review see Saga and Takeda, 2001). The authors concluded that a clock and a gradient are dispensable to form somites, and their role may be restricted to set their rostrocaudal polarity.

Dias et al. (2014) have shown that an epithelial tissue has the potentiality to spontaneously form physical boundaries by means of cell-cell interactions. However, the primary role of a clock-and-wavefront or any alternative model is not primarily to *create* boundaries, but to *periodically arrange* them, as this will contribute to the formation of a functional vertebral column. As the authors noticed, the ectopic somites are arranged as a bunch-of-grapes rather than being aligned. The question is, would these explants reproduce the *in vivo* periodicity of the PM if geometrically constrained? (Klumpers et al., 2014). In any case, Burgess et al. (1996) have shown that the PM is capable of segmenting into pieces even when epithelialization is inhibited, which indicates that there may exist more than one mechanism of PM segmentation.

## The Mechanical Approach

Some of the models previously discussed are based on features described in one taxon only. For example, ERK oscillations may set the timing of somite formation, but at present, they have only been described in the mouse embryo (Niwa et al., 2011). The same would apply to Wnt oscillations; conjointly with Notch oscillations, their phase shift can play a relevant role in the formation of somite boundaries, but they are only present in the mouse embryo (Sonnen et al., 2018). The presence of these oscillations in PM derived from human pluripotent cells *in vitro* suggests that they could also be important in human somitogenesis (Matsuda et al., 2020). The displacement of ERK activity in a stepwise manner can determine somite boundary at the uniform posterior PM, independently from the segmentation clock. However, this stepwise regression has only been observed in zebrafish (Naoki and Matsui, 2020). Although the main players of somitogenesis seem to be conserved among vertebrates (e.g., a maturation front and waves of gene expression) there appear to be relevant differences in their specific roles. Due to the possibility that PM progenitors could derive from different cell populations (Steventon and Martínez-Arias, 2017), different mechanisms could also underlie the formation of the anterior and posterior somites within a species (Shifley et al., 2008). This has probably contributed to the formulation of this wide range of hypotheses. In spite of this variety, all the models discussed above are based on the same idea: the existence of a gene regulatory network capable of forming a pre-pattern of gene expression, which guides PM morphogenesis. Below, we will discuss an alternative approach to somite periodicity.

### The Physics of Strain Softening

Alarcón et al. (2010) described the relationship between the cohesiveness of wetted, granular materials and the formation of cracks. The study was based on the notion that a small amount of water dramatically changes the mechanical properties of sand: dry sand runs through the fingers like a fluid, but wet sand can be shaped into castles and sculptures. Water creates weak, but numerous capillary bridges between the grains, which results in stickiness (cohesiveness) and a certain tensile strength that does not exist in fluid or dry sand. An analogous fluid-to-solid transition occurs in the PM by an increase of intercellular connections (Duband et al., 1987; Mongera et al., 2018). Alarcón et al. (2010) placed thin layers of wetted granules on an elastic substrate and subjected them to uniaxial tensile strain. This resulted in a periodic pattern of cracks that run perpendicularly to the direction of stretching. The tension applied on the granular layer opposes the capillary forces between the grains, and when a critical strain is reached, the connection breaks, a phenomenon called *strain softening*. Alarcón et al. (2010) further observed that cracks showed up at a characteristic mutual distance that appeared to be linearly dependent on: (a) the humidity of the sample; and (b) the thickness of the granular layer. Thus, a material needs to be cohesive (i.e., a solid rather than a fluid) in order to crack and the size of segments scales with the strength of cohesion and the thickness of the cracking body.

### Differential Strain in Embryogenesis

**FIGURE 2:**
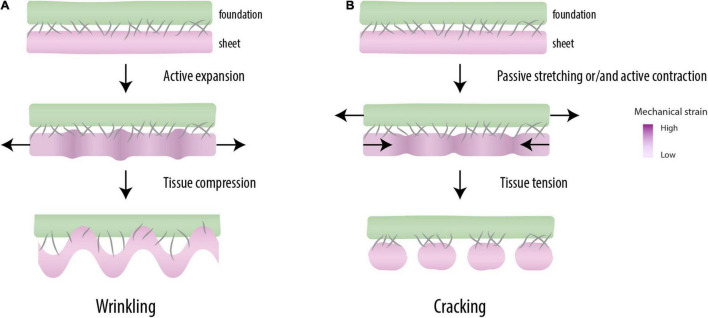
Mechanical patterning. When mutually adherent tissues expand at different rates (top), a periodic pattern of differential strain capable of driving morphogenesis is created (middle). When the mechanical instabilities are created under compression, one of the tissues forms wrinkles, a characteristic feature of the morphogenesis of some organs (e.g., guts, brain) (bottom) **(A)**. Under tension, these instabilities break up the tissue into regular pieces (cracking), as observed in somitogenesis (bottom) **(B)**.

The periodicity of cracks observed by Alarcón et al. (2010) is due to a phenomenon called *differential strain*: when two materials or tissues are physically connected (adherent) and one of them shrinks or extends with respect to the other, internal stresses are induced that cause mechanical instabilities capable of forming regular patterns (Figure 2). For example, Harris et al. (1984) showed that fibroblasts, when seeded uniformly on a gel connected to a glass fiber meshwork, eventually form a spatially periodic pattern of condensations. This is due to the contractility of the fibroblasts, which is resisted by a relatively stiff substrate (i.e., differential strain). Cell-seeded gels that are not mechanically restricted at their boundary do not show such periodicity, but become strongly contracted in their entirety (Bell et al., 1979; Klumpers et al., 2013). Spatial periodicity was also observed in the organization of pre-chondrogenic limb bud cells seeded on thin lines of fibronectin, which developed into a linear array of cellular condensations (Klumpers et al., 2014). The mutual distance of these condensations correlated to the width of the strip of fibronectin (Klumpers et al., 2014) and also appeared sensitive to external mechanical strain (Klumpers et al., 2015). These and other studies show that cells may self-organize into *periodic* multicellular structures by mechanical stress and that mechanical and geometrical constraints have morphogenetic potential (Harris et al., 1981; Harris, 2006; Mammoto and Ingber, 2010).

Differential strain in mutually adherent materials may result in two types of mechanical instability. Elastic materials that expand faster than a rigid underground compress themselves, which leads to geometric buckling phenomena like the wrinkling gut (Savin et al., 2011), the folded brain cortex (Tallinen et al., 2014), and the scoliotic spine (Crijns et al., 2017; Figure 2A). Tensile instabilities, the other type of mechanical instability, are related to the overstretching of a least elastic (i.e., most brittle) tissue in the construct (Figure 2B). In Harris et al. (1984) the contractile forces by the fibroblasts were large enough to tear the gel and the intercellular contacts in a regular geometric pattern. Harris (2006) argued that such mechanical instabilities can provide the cells with “positional information” (Wolpert, 1969) and thereby play a role normally attributed to morphogens. Tensile instabilities leading to periodic cracking are well known as surface crack patterns in inanimate materials, e.g., in crackle decorations (Zhao et al., 2011) and drying mud (Velde, 1999). More recently, they also have been related to morphological phenomena in organisms, like the crocodile skin (Milinkovitch et al., 2013).

Truskinovsky et al. (2014) made the case that also somite periodicity may be due to differential strain. With the presence of a contractile, coherent anterior PM and a physical connection to resistive surrounding tissues expanding at a different rates (like the ectoderm, the neural tube, the notochord or the intermediate mesoderm) (Bénazéraf et al., 2017; Marrese et al., 2020), the physical requirements for periodic differential strain are met. It is important to stress that the degree of tissue cohesiveness required for a pattern of differential strain to form is probably only met by the epithelium of the PM. This means that the segmentation of the mesenchyme could not result from the direct effect of differential strain. In a recent study, Nelemans et al. (2020) showed that a somite, when sufficiently strained, divides in two or more daughter somites under creation of a new boundary. The actin ring of the epithelial boundary was physically snapped, thereby opening the lateral side of the epithelial cells toward the mesenchymal cells in the somitocoel. This direct physical contact then leads to a lateral induction of epithelization (Kim et al., 2017), which creates the boundaries of the daughter somites. The boundaries were then made definitive by the deposition of fibronectin in between. Interestingly, Nelemans et al. (2020) did not observe new boundary formation in the posterior, more fluidic part of the presomitic PM, which confirms the notion that a certain level of cohesion is required for cracking to occur. Adhyapok et al. (2021) recently presented a computational model of periodic failure in the epithelial PM by mechanical strain, essentially describing the same mechanism of differential strain.

## Molecular and Mechanical Cues Are Involved in Somite Periodicity

According to molecular models, the segmentation program that determines somite boundary is autonomous (i.e., independent of surrounding tissues). This is in contrast with the mechanical model, in which the PM should be externally attached to be patterned. But is somite periodicity an intrinsic or an extrinsic process? There is experimental evidence suggesting that it is an intrinsic process. For example, an explant of PM cultured *in vitro* can form somites in isolation of surrounding tissues (Rifes et al., 2007). Some authors have suggested that the chemical patterning of the PM does not concomitantly lead to the formation of somite boundaries, which would require the presence of the surface ectoderm (i.e., an extrinsic factor) (Palmeirim et al., 1998). However, the role of the surface ectoderm would be mainly to provide the fibronectin matrix necessary to *stabilize* somite boundaries (Rifes et al., 2007), rather than mechanically pattern the PM. PM explants are usually placed on a filter paper floating in the culture medium, which would not meet the conditions of the differential strain hypothesis (i.e., the presence of a foundation) (Figure 2). In a similar way, cell aggregates of mouse embryonic stem cells can form trunk-like organoids with a high level of organization, which includes the formation of a neural tube, somites and a gut, but only if they are embedded in an extracellular matrix surrogate (Veenvliet et al., 2020).

Dray et al. (2013) have shown that in lack of integrin α5 and V5 in zebrafish embryos, molecules primarily involved in cell-fibronectin adhesion and fibronectin assembly, somites do not form. The authors have shown that this phenotype cannot be explained by alterations in cell migration, cell proliferation or cell differentiation, which seem to be similar to wild type embryos, but this would result from the loss of inter-tissue connections. The PM physically connects to surrounding tissues by fibronectin, in absence of integrin α5 and V5, this inter-tissue connections do not form, and the mechanical interactions between the PM and its surrounding tissues is impaired. In itgα5*^mo^*;αV*^mo^* embryos, a detached notochord undulates, as it would require to be mechanically coupled to the PM in order to grow straight. However, an alternative explanation for the lack of somites is the alteration of the fibronectin matrix covering the PM in these mutant embryos, as it should be intact for somite boundaries to form (Rifes et al., 2007).

According to the differential strain hypothesis, somite boundaries would form at locations of high strain at the epithelium, which would be periodically distributed throughout the PM. Sato et al. (2002) have shown that they can also form at regions of (predicted) low strain (i.e., within a somite). The authors transplanted posterior border cells (i.e., cells posterior to a mesenchymal forming-fissure) within a prospective somite in the presomitic PM. These cells induced the formation of an ectopic somite boundary, which seems to support the autonomy of the segmentation program. However, this experiment would not challenge the differential strain hypothesis if the transplant would contain an epithelial cleft committed to soften even under low stress (Shelton et al., 2021).

Burgess et al. (1996) studied the role of *paraxis* in somitogenesis and found that it is essential for epithelialization of the PM in the mouse embryo. When *paraxis* was mutated, the PM did not form epithelium, but the PM still segmented. As a difference from wild type embryos, however, the segmentation was irregular. This work reveals two important points: (1) the segmentation program is autonomous, i.e., the PM can split into pieces when patterning by differential strain is not possible, (2) inter-tissue connections would be required to form regular somites. Another observation in support of this view has been provided by Xiong et al. (2020). The authors incubated chicken embryos in which the adjacent tissues of the PM (i.e., the notochord and the neural tube) were dissected and their gap filled by a gel, i.e., inter-tissue connections where disrupted. Under this conditions the PM segmented, but the somites were larger and mislocated. Thus, inter-tissue mechanical coupling may be dispensable for segmentation, but may be necessary to refine the somite periodicity specified by a clock-and-wavefront mechanism.

Kunz et al. (2021) have demonstrated how the tension exerted by the vitelline membrane regulates the morphogenesis of the chicken embryo. During the first day of development, the tension of the vitelline membrane is necessary for the extension of the blastoderm. Later on, downregulation of this tension allows the formation of the body axis. Inhibition of this downregulation leads to the widening of the neural tube and the PM, a reduction of body elongation, and in some cases, an open neural tube. Remarkably, some of these embryos form a reduced number of somites, a phenotype that may result in sacral agenesis (Postma et al., 2014). Thus, mechanical forces do seem to play a role in the early developmental stages of spine development.

## Discussion

Based on the literature reviewed in the present work, it seems reasonable to think that molecular and mechanical cues would *conjointly* determine somite periodicity. We suggest that the epithelium of the PM is mechanically patterned by differential strain, and that this contributes to the formation of regular somites by fine tuning MESP expression. It is important to stress that molecular models do not specify whether the MESP signal is restricted to the mesenchyme or it is also expressed in the epithelium. In general, *in situ* hybridization of PM genes are not performed at the resolution necessary to check this relevant aspect of morphogenesis. From some images of high-resolution *in situ* hybridization, it seems that *mesp2* could be restricted to the mesenchyme in the mouse embryo (see Figure 2 in Oginuma et al., 2010). However, even if the clock-and-wavefront mechanism patterns the epithelium, an additional mechanism would be necessary to explain why epithelial clefts are visible several somite lengths caudal to the formation of a mesenchymal fissure (Bellairs, 1979; Meier, 1979; Adhyapok et al., 2021).

**FIGURE 3:**
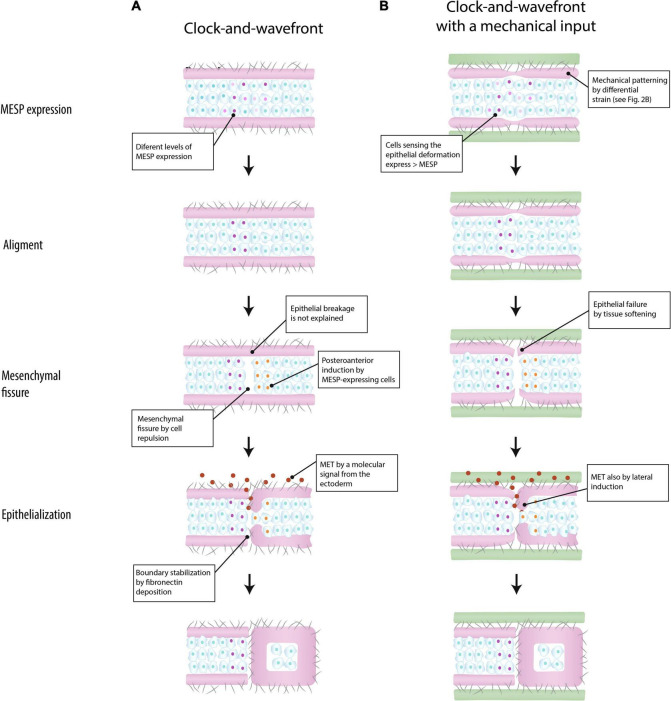
Models of somite boundary specification in the paraxial mesoderm. According to Takahashi and Sato (2008), a somite boundary is marked by a clock-and-wavefront mechanism that triggers the expression of the segmentation program (MESP expression) in the mesenchyme. The epithelium plays a passive role **(A)** (based on Takahashi and Sato, 2008). In the integrated model suggested in the present work, the epithelium is mechanically patterned by differential strain, and conjointly with a clock-and-wavefront, it marks the location of somite boundary by aligning MESP expression (see the section “Discussion” for details) **(B)** (green: surrounding tissue; purple: epithelium; cells: mesenchyme; gray fibers: extracellular matrix; MET: mesenchymal-to-epithelial transition).

Figure 3A shows how a new somite boundary is formed according to Takahashi and Sato (2008). Initially, the location of a new boundary is roughly defined by MESP-expressing cells triggered by a clock-and-wavefront mechanism. Subsequently, those located at the ventral side behave like organizers that induce the alignment of MESP-expressing cells along the ventro-dorsal axis. Once MESP-expressing cells are aligned, they induce the formation of the mesenchymal fissure by regulating cell repulsion, e.g., *via* Ephrin signaling. Finally, a molecular signal from the surface ectoderm penetrates into the fissure and induces the epithelialization of the posterior border cells previously patterned by MESP-expressing cells, thereby forming a new somite boundary. This last step would require the epithelium breakage at the level of the mesenchymal fissure, a step that is not explained by this model.

In the proposed integrated view, the epithelium plays a relevant role in somite periodicity. This tissue layer would be mechanically patterned by inter-tissue connections about four somite-lengths caudal to the formation of a mesenchymal fissure (Figure 3B). Like in the anterior model (Figure 3A), MESP-expressing cells roughly determine the location of the next somite boundary and they are aligned along the ventro-dorsal axis by the ventral cells. But in addition, these coordinating cells are induced by the mechanical deformation at the epithelial layer. The idea is that mesenchymal cells closer to the epithelial cleft will sense this mechanical deformation and respond by expressing higher levels of MESP. In the mouse embryo, this mechanotransduction could be mediated by Yap signaling (Hubaud et al., 2017), as this pathways seems to be linked to the segmentation clock. Once they are aligned, MESP-expression cells will induce a fissure in the mesenchyme *via* cell repulsion, at the same time that the epithelial layer will break up by tissue softening. The epithelialization of the posterior border cells could be induced by either a signal from the ectoderm or by direct contact with detached epithelial cells (Kim et al., 2017). According to this model, somite boundaries will still form in absence of inter-tissue connections, but in a less regular way, as observed *in vivo* (Burgess et al., 1996; Xiong et al., 2020).

This integrated model could help to better understand somite scaling. As previously commented, somite size consistently scales with the length of the presomitic mesoderm (Ishimatsu et al., 2018). How this is achieved is a major question in the field (Cotterell et al., 2015; Nesterenko and Zaraisky, 2019; Naoki and Matsui, 2020). Some workers have provided molecular models to explain scale invariance in somitogenesis (Lauschke et al., 2013; Umulis and Othmer, 2013; Cotterell et al., 2015; Nesterenko and Zaraisky, 2019; Naoki and Matsui, 2020; Clark, 2021). Here we suggest that differential strain may be another scaling mechanism at work in the developing embryo. Periodic cracking intrinsically scales with the size of the object (Alarcón et al., 2010; Thouless et al., 2011; Truskinovsky et al., 2014), which is due to the linear relation between the thickness of the cracking substance and the stresses induced by differential strain. That is, a bigger object will break into larger pieces. This means that differences in the size of the PM would *automatically* lead to changes in the position of foci of mechanical strain, which in turn could fine tune somite periodicity.

Although Nelemans et al. (2020) showed that new boundaries can form in existing somites under high stress, at present, there is no experimental evidence in support of differential strain in the presomitic PM. A step forward to this aim would be to test if the clefts of the epithelial layer are absent when inter-tissue connections are disrupted. This disruption can be performed *in vivo* as described by Xiong et al. (2020) and Kunz et al. (2021) and the presence or absence of clefts in the treated embryos can be tested by electron microscope. If foci of high strain play a role in the alignment of MESP-expression cells, in their absence, it is expected MESP stripes would be less defined. If this is confirmed, the next step would be to determine if MESP expression can be fine-tuned by the deformation of the epithelium. The link between Yap signaling and the segmentation clock could explain this alignment, but it is primarily based on a single study in the mouse embryo (Hubaud et al., 2017). Probably this mechanism differs among taxa.

Regarding the role of Yap in somitogenesis, Hubaud et al. (2017) have reported the lack of a qualitative change in the localization of YAP1 between posterior cells and those that slow down their clocks in the mouse embryo, as it would be expected if the clock is regulated by a mechanical input *via* Yap signaling. However, this difference does not necessarily need to be qualitative, but it may also be quantitative: the involvement of Yap signaling in developmental processes is frequently measured as a change in the nucleus-to-cytoplasm ratio (e.g., Shreberk-Shaked and Oren, 2019). That is, the lack of an obvious, qualitative difference in the localization of YAP1 between the posterior and the anterior PM does not rule out the possibility that the clock may be mechanically regulated in the mouse embryo.

The differential strain hypothesis could be directly tested *in vitro* by generating internal stress to an epithelial layer attached to a substratum (Figure 3B). It would be expected that this internal stress will form regularly spaced cracks in the tissue, analogous to those formed in drying mud or wetted granular materials. The cell line used to form this epithelial layer would not be relevant, as the hypothesis theoretically applies to any kind of coherent tissue. By adding a layer of mesenchymal cells underneath the epithelial layer, it could be tested if these cracks are capable to segment the whole construct. If confirmed, this could represent an ancestral mechanism of PM segmentation. In a growing embryo in which physically connected tissues are expanding at different rates (Bénazéraf et al., 2017), the differential strain hypothesis predicts that, under certain conditions, the periodic breakup of a tissue would be unavoidable. From this view, embryonic tissues would be able to form periodic structures *spontaneously*, without the need for any chemical pre-pattern, if only they are able to soften under mechanical strain (Alarcón et al., 2010). Strain softening has been reported in cultured cells (Puech et al., 2005) and in zebrafish somitogenesis (Shelton et al., 2021). Shelton et al. (2021) have shown that a stress localization in the solid-like, anterior PM of a zebrafish induces a fluidization of cells adjacent to the forming somite border. This type of strain softening adds a cytoskeletal component of actin deposition and its activation by myosin, thereby creating a feedback loop of increasing tension that culminates in fluidization and separation. Tissue patterning by differential strain could serve as a template for the evolution of divergent gene regulatory networks, as those involved in a clock-and-wavefront mechanism (Krol et al., 2011).

As reflected by the number of paragraphs dedicated to each approach, molecular studies have received most of the attention in the study of somite periodicity. Here we argue that mechanical patterning by differential strain is a feasible hypothesis that could be integrated with a clock-and-wavefront mechanism. The proposed model would link the segmentation of the PM with the morphogenesis of the surrounding tissues, a missing aspect in available molecular models. This mechanical input could contribute to the robustness of the PM segmentation. Furthermore, differential strain would constitute a physical principle applicable to somite periodicity across taxa, independently of differences at the molecular level. We hope the present review could stimulate future work on the mechanical patterning of the PM.

## Author Contributions

ML-M and THS conceived and wrote the manuscript. Both authors contributed to the article and approved the submitted version.

## Conflict of Interest

The authors declare that the research was conducted in the absence of any commercial or financial relationships that could be construed as a potential conflict of interest.

## Publisher’s Note

All claims expressed in this article are solely those of the authors and do not necessarily represent those of their affiliated organizations, or those of the publisher, the editors and the reviewers. Any product that may be evaluated in this article, or claim that may be made by its manufacturer, is not guaranteed or endorsed by the publisher.

## Supplementary Material

Supplementary Movie 1 Segmentation of the paraxial mesoderm in the chicken embryo from a dorsal view (anterior is left, and posterior is right) (by Nelemans and Schmitz).
